# Uncovering the Underlying Mechanisms of Ketamine as a Novel Antidepressant

**DOI:** 10.3389/fphar.2021.740996

**Published:** 2022-07-07

**Authors:** Songbai Xu, Xiaoxiao Yao, Bingjin Li, Ranji Cui, Cuilin Zhu, Yao Wang, Wei Yang

**Affiliations:** ^1^ Department of Neurosurgery, First Hospital of Jilin University, Changchun, China; ^2^ Jilin Provincial Key Laboratory on Molecular and Chemical Genetic, The Second Hospital of Jilin University, Changchun, China; ^3^ Department of Cardiovascular Surgery, The Second Hospital of Jilin University, Changchun, China

**Keywords:** major depressive disorder, antidepressant, ketamine, NMDAR, BDNF, mTOR

## Abstract

Major depressive disorder (MDD) is a devastating psychiatric disorder which exacts enormous personal and social-economic burdens. Ketamine, an *N*-methyl-D-aspartate receptor (NMDAR) antagonist, has been discovered to exert rapid and sustained antidepressant-like actions on MDD patients and animal models. However, the dissociation and psychotomimetic propensities of ketamine have limited its use for psychiatric indications. Here, we review recently proposed mechanistic hypotheses regarding how ketamine exerts antidepressant-like actions. Ketamine may potentiate α-amino-3-hydroxy-5-methyl-4-isoxazole-propionic acid receptor (AMPAR)-mediated transmission in pyramidal neurons by disinhibition and/or blockade of spontaneous NMDAR-mediated neurotransmission. Ketamine may also activate neuroplasticity- and synaptogenesis-relevant signaling pathways, which may converge on key components like brain-derived neurotrophic factor (BDNF)/tropomyosin receptor kinase B (TrkB) and mechanistic target of rapamycin (mTOR). These processes may subsequently rebalance the excitatory/inhibitory transmission and restore neural network integrity that is compromised in depression. Understanding the mechanisms underpinning ketamine’s antidepressant-like actions at cellular and neural circuit level will drive the development of safe and effective pharmacological interventions for the treatment of MDD.

## 1 Introduction

Major depressive disorder (MDD), characterized by increased negative affect (depressed mood) and reduced positive affect (anhedonia), is the most prevalent psychiatric disorder in the modern world (Collins et al., 2011; Otte et al., 2016). So far, standard antidepressant medications include tricyclic antidepressants (TCAs), serotonin selective reuptake inhibitors (SSRIs), and monoamine oxidase inhibitors (MAOIs). When administrated alone or in combination, these antidepressants suffer with high rates of partial- or non-response, limited duration of efficacy, and a significant delay in the onset of therapeutic action. Such shortcomings of traditional antidepressants have hindered their application to patients with resistance to standard treatment or with suicidal ideation which demands immediate intervention (Al-Harbi, 2012; Rincon-Cortes and Grace, 2020). The serious health and socio-economic burdens caused by unsuccessfully managed depression have driven the researches for new pharmacotherapeutic interventions overcoming such limitations.

Ketamine, a non-competitive *N*-methyl-D-aspartate receptor (NMDAR) antagonist, has demonstrated a rapid and sustained antidepressant-like effect with a single administration of sub-anesthetic dose in treatment-resistant (refers to an inadequate response of patient to at least two different antidepressants administered at adequate doses and duration) depressed patients. Although bearing such merits, the clinical application of ketamine as an antidepressant has been restricted due to its abuse potential and dissociation properties. A full understanding about the exact mechanisms of the antidepressant-like action of ketamine will provide invaluable insights on the neurobiology of depression, and foster the development of rapid-acting antidepressants without side effects (Kavalali and Monteggia, 2015; Monteggia and Zarate, 2015; Krystal et al., 2019; Jelen et al., 2021). In this review, we will present recently proposed mechanisms about how ketamine exerts antidepressant-like action. These hypotheses include disinhibition of glutamate transmission,inhibition of NMDAR-mediated transmission at rest, activation of BDNF/TrkB and mTOR signaling pathways. Subsequently, these processes may synergistically promote synaptogenesis and enhance synaptic plasticity. Sustained excitatory/inhibitory neurotransmission balance and restored neural network integrity may be critical factors contributing to the antidepressant-like effects of ketamine.

## 2 Ketamine and its Metabolites as Antidepressant Drugs

Ketamine is a racemic mixture of equal amounts of two enantiomers, (*S*)-ketamine and (*R*)-ketamine. After administration, ketamine has been reported to exert anesthetic, analgesic, anti-inflammatory, and antidepressant effects. The anesthetic and analgesic properties of ketamine are generally attributed to direct inhibition of NMDARs, whereas the exact mechanism of its antidepressant-like action remains a hot issue under debate (Zanos et al., 2018a).

**FIGURE 1:**
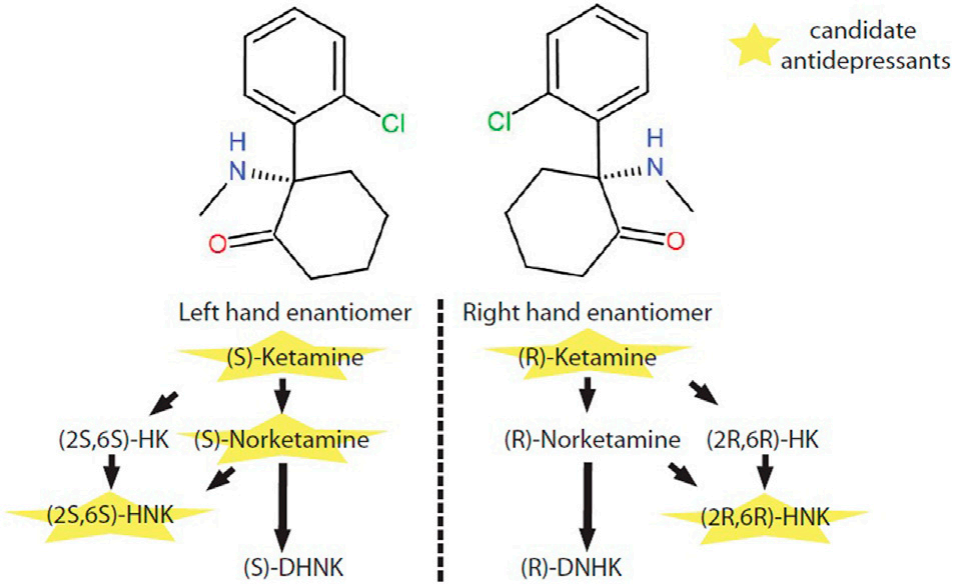
Metabolic pathway of ketamine enantiomers. (S)-ketamine and (*R*)-ketamine are a pair of stereoisomers. *In vivo*, (*S*)-ketamine and (*R*)-ketamine is initially demethylated to (*S*)-norketamine or (*R*)-norketamine. (*S*)-norketamine or (*R*)-norketamine is further metabolized to (*S*)-DHNK or (*R*)-DHNK. (*S*)-norketamine or (*R*)-norketamine can also be hydroxylated into (*2S,6S*)-HNK or (*2R,6R*)-HNK, respectively. Recently, ketamine and several of its metabolites with potential antidepressant-like effects have intrigued enthusiastic investigations at preclinical and clinical levels. Abbreviations: (*2R*,*6R*)-HK, (*2R*,*6R*)-hydroxyketamine; (*2S*,*6S*)-HK, (*2S*,*6S*)-hydroxyketamine; (*2R*,*6R*)-HNK, (*2R*,*6R*)-hydroxynorketamine; (*2S*,*6S*)-HNK, (*2S*,*6S*)-hydroxynorketamine; (*R*)-DHNK, (*R*)-dehydronorketamine; (*S*)-DHNK, (*S*)-dehydronorketamine.

*In vivo*, (*S*)-ketamine or (*R*)-ketamine is firstly demethylated to (*S*)-norketamine or (*R*)-norketamine. (*S*)-norketamine is subsequently metabolized to (2*S*,6*S*)-hydroxynorketamine (HNK) or (*S*)-dehydronorketamine (DHNK). Accordingly, (*R*)-norketamine is subsequently metabolized to (2*R*,6*R*)- HNK or (*R*)- DHNK. (*S*)-ketamine and (*R*)-ketamine can also be metabolized into (2*S*,6*S*)-hydroxyketamine (HK) and (2*R*,6*R*)-HK, which are further transformed to (2*S*,6*S*)-HNK and (2*R*,6*R*)-HNK respectively (Jelen et al., 2021) (Figure 1).

In 2000, a pilot clinical trial reported that sub-anesthetic dose of ketamine, when administrated intravenously, exerted a robust antidepressant-like effect within 4 h post infusion (Berman et al., 2000; Zanos and Gould, 2018). This discovery has since intrigued a series of studies investigating the potential of ketamine as a candidate antidepressant. Randomized, double-blind clinical trials validated the efficacy of ketamine in treatment-resistant major depression (TRD) (Zarate et al., 2006; Murrough et al., 2013; Lapidus et al., 2014; Singh et al., 2016). These studies consistently reported a rapid and robust onset of the antidepressant-like action of ketamine within several hours post administration, which may benefits cases of patients at imminent risk of suicide. As a proof of this concept, ketamine has been reported to rapidly ameliorate suicidal ideation in major depressed patients and to rapidly reduce anhedonia (Price et al., 2009; Ballard et al., 2014; Lally et al., 2014). Moreover, prolonged antidepressant-like effects of ketamine have been documented, adding another layer of glamour to this potential antidepressant. For instance, a clinical study reported that in 35% of depressive patients, a single intravenous dose of ketamine exerted a sustained antidepressant effect which lasted for up to 1 week (Zarate et al., 2006).

In preclinical studies, (*R*)-ketamine shows a greater potency and longer-lasting antidepressant-like effect than (*S*)-ketamine (esketamine) (Bonaventura et al., 2021). In behavioral tests for side effects, (*S*)-ketamine, but not (*R*)-ketamine, precipitated behavioral abnormalities, such as hyperlocomotion and prepulse inhibition deficits (Yang et al., 2015). Early pharmacodynamic studies demonstrated that ketamine and norketamine exerted anesthetic effects and induced hyper-locomotor activity during recovery period in rats, whereas HNK lacked such effects. HNK was thus described as “inactive” metabolite in regard to ketamine’s anesthetic action (Singh et al., 2014). Interestingly, it has been reported independently by several groups that metabolism of ketamine to HNK is necessary for its antidepressant-like action in rodents (Zanos et al., 2016; Pham et al., 2018; Fukumoto et al., 2019) (but see Yang et al., 2017; Grunebaum et al., 2019; Farmer et al., 2020 for arguments against this conclusion). Collectively, the reported characteristics of ketamine and its metabolites suggested a possibility that the anesthetic and antidepressant-like action might be separated and be exploited for the development of novel antidepressants.

## 3 Prefrontal Cortex as an Important Region Mediating the Antidepressant-Like Effects of Ketamine

As a central hub that receives input from cortical, thalamic, and limbic regions and sends outputs to structures that regulate emotion, fear, and stress responses, the PFC has been demonstrated to be highly involved with emotional and cognitive processing, and to play a critical role in mood disorders, such as anxiety, depression, and schizophrenia. Previous studies reveal that the PFC is necessary and sufficient for the actions of ketamine (Lepack et al., 2014; Fuchikami et al., 2015; Bittar and Labonte, 2021). Inactivation of the medial FPC (mPFC), a subregion of PFC, completely blocked the antidepressant and anxiolytic effects of systemic ketamine in rodent models. On the other hand, ketamine microinfusion into the mPFC reproduced the behavioral action of systemic ketamine (Fuchikami et al., 2015). It is hypothesized that ketamine exerts antidepressant-like effects by rebalancing the neural network activity in the PFC and its downstream targeting regions.

### 3.1 Ketamine May Normalize the Network Activity and Connectivity in the Prefrontal Cortex

Both animals and human beings are challenged with a variety of physical and psychological problems on a daily basis. It is theorized that distinct networks are dynamically recruited to fulfill various tasks. The default mode network (DMN) is associated with introspection. The salience network (SAL) processes salient information from external sources, and the central executive network (CEN) participates in working memory and attention. Studies have demonstrated an increase in the activity of the DMN and a decrease in SAL and the CEN in depressed patient. These alterations may increase the time patient spend in rumination or introspection, and impair their ability to deploy attentions to tasks that require responses to external stimuli (Greicius et al., 2007; Kaiser et al., 2015; Evans et al., 2018).

In healthy subjects, ketamine decreases functional connectivity of the DMN to the mPFC (Scheidegger et al., 2012), thus may disengage the mPFC from DMN in introspection status. In MDD patients, ketamine normalizes disconnectivity between the PFC and the rest of the brain (Abdallah et al., 2017; Kraus et al., 2020), and correlation analysis suggests that connectivity changes might be used as a prediction of subject’s response to the antidepressant effect of ketamine. In the PFC of rodents, ketamine increases gamma-band power, a putative measure of cortical disinhibition and an indicator of fast ionotropic excitatory receptor activation (Muthukumaraswamy et al., 2015).

These studies suggest that ketamine might exert its antidepressant-like effects by normalizing the PFC network activity and connectivity. Meanwhile, cortical electrophysiological measurement via non-invasive approaches may serve as a biomarker to evaluate the effectiveness of the antidepressant-like action of ketamine. However, more invasive methodologies on animal models are necessary to delineate the mechanism of ketamine’s antidepressant-like action at molecular level.

### 3.2 Prefrontal Cortex Afferents Target Brain Regions Implicated in Depression

With the advancement of optogenetic and chemogenetic techniques, accumulating evidences indicate that several brain regions functionally connected with the PFC are highly involved with mood disorders including anxiety and depression. Ketamine may exert antidepressant-like effects *via* modulation of activity and plasticity in these neural circuitries. We will briefly discuss recent discoveries corroborating this hypothesis in the following paragraph.

In a learned helplessness paradigm model, a single dose of ketamine successfully rescued escape behavior in mice. Although fiber photometry and chemogenetic inhibition demonstrated that dopaminergic neuron activity in the ventral tegmental area (VTA) is necessary for the behavioral effects of ketamine, the authors suggested against a direct cell-autonomous regulation of VTA dopaminergic neurons by ketamine. Further experiments revealed that ketamine increased population activity in D1R-expressing pyramidal neurons in the mPFC (Marcus and Bruchas, 2021, Wu et al., 2021b). These neurons synapsed onto VTA dopaminergic neurons, thus indirectly altered the responses of VTA dopaminergic neurons in escape behavior. CCK administration into mPFC mimics the anxiogenic- and depressant-like effects of social stress in mice. Optogenetical stimulation of mPFC to basolateral amygdala (BLA) projection blocked the anxiogenic effect of CCK, whereas optogenetical stimulation of the mPFC to nucleus accumbens (NAc) reversed CCK-induced social avoidance and sucrose preference deficits (Vialou et al., 2014). Optogenetic stimulation of the projection from the infralimbic cortex (IL), a subregion of the mPFC, to lateral septum (LS) promoted anxiety-related behaviors, whereas activation of the projection from the IL to the central nucleus of the amygdala (CeA) exerted anxiolytic and effects (Chen et al., 2021). Optogenetic stimulation of the projection from the mPFC to the dorsal raphe (DRN) significantly increased kick frequency in forced swimming test in rat, indicating an antidepressant response. On the contrary, activation of the projection from the mPFC to the lateral habenula (LHb) decreased mobility in forced swimming test, indicating opposite roles of the mPFC-to-DRN and mPFC-to-LHb neural pathways in driving motivated behavioral response to a challenging environment (Warden et al., 2012). In forced swim test, pharmacological and optogenetic inactivation of the ventral hippocampus (vHipp) prevented the sustained antidepressant-like effect of ketamine. On the contrary, optogenetic and chemogenetic activation of the vHipp-mPFC pathway mimicked the antidepressant-like response to ketamine in a pathway specific manner (Carreno et al., 2016). Optogenetic activation of *Drd1*-expressing pyramidal cells in the mPFC mimicked rapid and sustained antidepressant-like effects of ketamine in behavioral responses. On the contrary, local infusion of D1R antagonist, optogenetic inhibition and chemogenetic inhibition of *Drd1*-expressing pyramidal neurons in the mPFC abolished the antidepressant-like actions of systemic ketamine. Stimulation of mPFC Drd1 terminals in the BLA recapitulates the antidepressant-like effects of somatic stimulation in the mPFC (Hare et al., 2019).

To summarize, these studies suggest that several brain regions, including VTA, NAc, BLA, CeA, vHipp, DRN, LHb, PAG, and LS, are directed targeted by PFC afferents and are implicated in depressive-like behaviors, and these regions may employ adaptive changes by antidepressant dose of ketamine. Although it is generally acknowledged that ketamine increases the network activity and induces glutamate surge (will be discussed later) in the mPFC, optogenetic and chemogenetic activation of the downstream targets of the PFC leads to different behavioral outcomes (anxiolytic vs. anxiogenic) depending on the particular circuit investigated. How a unanimous excitation in the PFC, generated by systemic ketamine administration, is translated into distinct activity profiles in different brain regions receiving reciprocal projection from the PFC? Since the total behavioral outcome of ketamine administration is antidepressive-like, does is mean the activation of anxiolytic regions can dominate and successfully antagonize the activation of anxiogenic regions under the influence of ketamine? Or other mechanisms are also involved in remodeling the activity of brain network by ketamine? Answering these questions at neural circuit level will deepen our understanding about the etiology of depression.

## 4 Mechanisms of How Ketamine Exerts Antidepression-Like Effects

### 4.1 Disinhibition of Glutamate Transmission by Targeting GABAergic Interneurons

**FIGURE 2:**
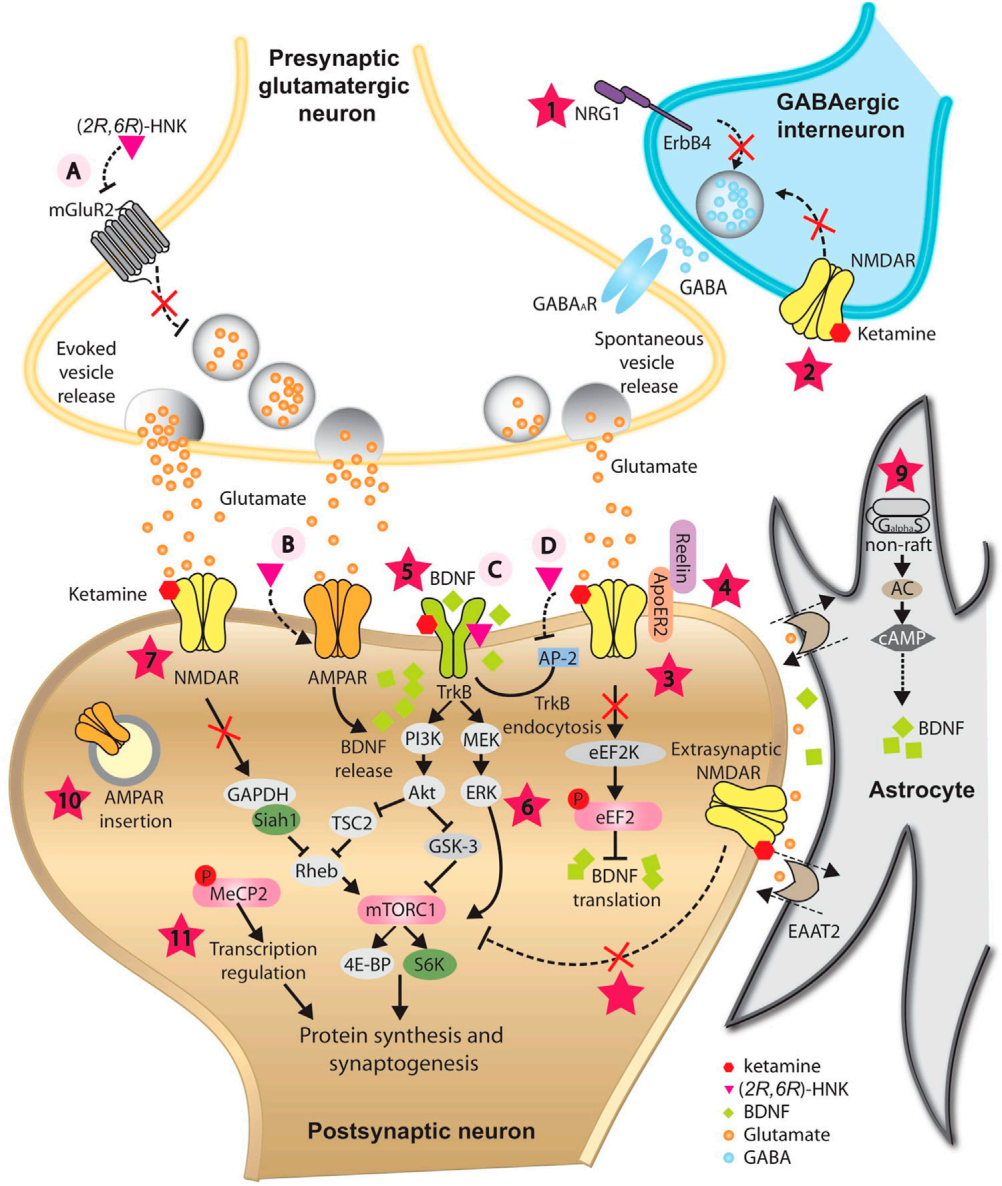
Proposed mechanisms underlying the antidepressant-like effects of ketamine and its metabolite (*2R*,*6R*)-HNK. In GABAergic inhibitory interneurons, ketamine is proposed to decrease GABA release onto glutamatergic presynaptic neurons by antagonizing NRG1/ErbB4 signaling pathway **(1)** and/or blockade of NMDARs **(2)**, resulting in disinhibition of pyramidal neurons. Augmented glutamate transmission activates AMPARs and subsequently enhances BDNF release. In postsynaptic neurons, ketamine is proposed to augment BDNF/TrkB pathway by releasing the inhibition of BDNF translation from eEF2K **(3)**, directly binding with its receptor TrkB **(5)**, and/or by activating the PI3K-Akt and MEK-ERK signaling pathways **(6)**. One of the downstream targets activated *via* PI3K-Akt and MEK-ERK signaling pathways is mTORC1, which is implicated in synaptogenesis- and synaptic plasticity-relevant processes. Besides direct activation by BDNF/TrkB signaling, blockade of NMDARs by ketamine is proposed to activate mTORC1 indirectly through compromising the ubiquitin-mediated degradation of mTORC1 activator Rheb **(7)**, or by inhibition of extrasynaptic NMDARs **(8)**. At rest, spontaneous vesicle release may play an important role in maintaining the activation of NMDARs at basal level. Reelin/ApoER2 may be implicated in this process and be modulated by ketamine **(4)**. Inhibition of NMDAR by ketamine blockade may promote AMPAR insertion at postsynaptic sites as a homeostatic process **(10)**. BDNF/TrkB signaling pathway may trigger phosphorylation of MeCP2, which is essential for sustained antidepression-like action of ketamine **(11)**. In astrocyte, ketamine is proposed to trigger the translocation of Gas to non-raft domains, leading to enhanced AC activity and elevated BDNF expression **(9)**. (*2R*,*6R*)-HNK may act on the presynaptic terminal to increase glutamate release, possibly *via* inhibition of mGluR2 receptors **(A)**. Elevated glutamate release may activate postsynaptic AMPARs and enhance BDNF release **(B)**. (*2R*,*6R*)-HNK may also facilitate the BDNF/TrkB signaling pathway by directly binding with TrkB **(C)**, or by disrupting the interaction between TrkB and AP-2, thus inhibiting the endocytosis of TrkB **(D)**. These mechanisms may have different temporal spatial features and may function synergistically or complementarily, leading to a rebalanced excitatory/inhibitory neurotransmission and restored integrity of neural circuits, which is indispensible for the antidepressant-like action of ketamine and (*2R*,*6R*)-HNK.

Ketamine was at first identified as an *N*-methyl-D-aspartate glutamate receptor (NMDAR) antagonist (Figure 2). Recently, a detailed structural analysis revealed the mechanisms by which ketamine inhibits NMDA receptors. Both hydrogen-bond (on GluN1 subunit) and steric-sensitive hydrophobic interactions (on GluN2 subunit) are essential to stabilize the binding of ketamine enantiomers. These principles apply to ketamine’s binding with both GluN1–GluN2A and GluN1–GluN2B NMDA receptors. Structural comparison among ionotropic glutamate receptors suggested that amino acid differences in key sites may interfere with the binding of ketamine, and may dictate the selectivity of ketamine for NMDA receptors over AMPA and kainate receptors. On the other hand, the hydroxyl group on cyclohexane in HNK may disrupt the hydrophobic interaction with GluN2 subunits, rendering HNK a much less potent antagonist for NMDAR blockade (Zhang et al., 2021a).

It has been repetitively verified that one of the mechanisms underlying the rapid antidepressant effects of ketamine is through blockade of NMDARs on GABAergic inhibitory interneurons in the PFC, which leads to a rapid increase glutamatergic release and activation of post synaptic AMPARs (Henley and Wilkinson, 2016; Krystal et al., 2019). A subanesthetic dose of ketamine significantly increases glutamate cycling (Chowdhury et al., 2017) and presynaptic glutamate release (Moghaddam et al., 1997) in the PFC. As a consequence of augmented glutamate neurotransmission, ketamine increases overall excitability of the PFC in healthy volunteers and animal models (Zanos and Gould, 2018; Zhang et al., 2021a). Additionally, ketamine enhances gamma band electroencephalography power, which is closely related with cortical disinhibition (Hong et al., 2010).

Perfusion of ketamine significantly diminishes the frequency of action potential in fast spiking interneurons in the mPFC, and pharmacological inhibition of the parvalbumin (PV) neurons in the mPFC abolishes the antidepressant-like effects of ketamine in behavioral animal models (Zhang et al., 2021a). Subanesthetic ketamine rapidly suppresses the activity of somatostatin (SST) interneurons in the PFC. This loss of dendritic inhibition by SST neurons augments evoked synaptic calcium transients in the apical dendritic spines of pyramidal neurons (Ali et al., 2020). In the mPFC, virus-mediated knockdown of the GluN2B subunit of NMDA receptor in GABAergic interneurons blocks ketamine-induced disinhibition of principle neurons, and occludes the antidepressant-like actions of ketamine in behavioral animal models (Gerhard et al., 2020).

The preferential action of ketamine at inhibitory interneurons is postulated to be due to higher frequency of interneuron firing compared with pyramidal neurons, which allows for depolarization-dependent relief of Mg^2+^ blockade and exposure of binding site for ketamine at NMDAR channel pore selectively (Zanos and Gould, 2018). Ketamine may selectively inhibit certain subunits of NMDARs predominantly expressed in interneurons. For instance, at physiological extracellular Mg^2+^ concentration, ketamine is reported to have higher affinity for NMDARs containing GluN2D subunit, which are abundantly expressed in forebrain interneurons (Monyer et al., 1994; Perszyk et al., 2016). This propensity may also contribute to the observed preference of inhibition on GABAergic transmission by ketamine.

#### 4.1.1 Subunit Selectivity of Ketamine’s Binding With *N*-Methyl-D-Aspartate Receptors

The four types of GluN2 subunits (GluN2A-GluN2D) determine many important characteristics of the NMDARs, including channel open probability, single-channel conductance, pharmacological sensitivities, and deactivation rate. Channel blockade by extracellular Mg^2+^ is much weaker for receptors containing GluN2C and GluN2D subunits (Glasgow et al., 2015). Expression and subcellular localization of NMDAR subunits varies by developmental stage, brain region and cell type.

Studies have indicated that GluN2A subunits are expressed preferentially at synaptic sites, whereas GluN2B subunits are expressed preferentially at extrasynaptic sites (Hardingham and Bading, 2010; Paoletti et al., 2013; Parsons and Raymond, 2014). Recently, it is reported that inhibition of NMDAR by ketamine depends on the duration of glutamate exposure and the compositions of NMDAR (Glasgow et al., 2017; Glasgow et al., 2018). Moreover, recovery from desensitization of GluN1/2B receptors is accelerated by ketamine, whereas the desensitization of GluN1/2A receptors is unchanged. On the contrary, another group reported that ketamine acts indistinguishably at synaptic and extrasynaptic NMDARs in cultured hippocampal neurons (Emnett et al., 2013). This report posits that ketamine binds with NMDARs in an unbiased manner disregarding subunit composition of NMDARs. The discrepancy may originate from different experimental condition such as cell types and concentrations of drugs applied. Distinct location and distribution of NMDAR subunits may lead to diverse activation profiles depending on the concentration and duration of glutamate they sense, and thus be differently modulated by ketamine, depending on factors that impact synaptic NMDAR channel opening. These factors may include glutamate release-probability, subunit composition of synaptic NMDARs, as well as levels of co-agonists.

Channel blockade by extracellular Mg^2+^ is much weaker for receptors containing GluN2C and GluN2D subunits (Glasgow et al., 2015). This property renders receptor containing GluN2C and GluN2D subunits more susceptible to ketamine inhibition by competing with physiological concentration of Mg^2+^. Inhibition of NR2D-containing NMDARs could selectively reduce excitation of a subset of inhibitory neurons that highly express the NR2D subunit (Monyer et al., 1994). It has been suggested that ketamine preferentially act on GluN2C and/or GluN2D-containing NMDARs at a concentration that has psychotogenic action in humans (Kotermanski and Johnson, 2009; Khlestova et al., 2016).To support this hypothesis, the psychotomimetic effect of ketamine is abolished in GluN2C knockout mice, while the antidepressant-like response to ketamine is fully reserved (Tarres-Gatius et al., 2020). This observation suggests that GluN2C subunit may discriminate between antidepressant-like and psychotomimetic action of ketamine, and this feature may be exploited to screen new fast-acting antidepressant compounds devoid of propsychotic action and abuse potential.

#### 4.1.2 Neuregulin1/ErbB4 Signaling Pathway

NRGs comprise a large family of growth factors that stimulate ErbB receptor tyrosine kinases. NRG/ErbB signaling has been implicated in neural development processes, including circuit generation, axon ensheathment, neurotransmission and synaptic plasticity. NRGs and their receptors ErbBs have been identified as susceptibility genes for psychiatric disorders such as schizophrenia, bipolar disorder and depression (Deng et al., 2013) (Mei and Nave, 2014; Sun et al., 2016; Fiori et al., 2020; Grieco et al., 2021).

NRG and ErbB kinases, in particular ErbB4, are indispensible for the assembly of GABAergic circuit (Mei and Nave, 2014; Sun et al., 2016). Ketamine decreases the levels of NRG1 and phosphorylated ErbB4 (Wang et al., 2014), and rapidly downregulates NRG1/ErbB4 signaling in the mPFC(Grieco et al., 2021). Both studies reported a net effect of rapid and sustained cortical disinhibition, as reflected by elevated glutamate level, increased calcium activity and increased phosphorylation of CREB at Ser133. The disinhibition effect of ketamine is abolished by ErbB4 receptor knockout selectively in PV interneurons.

To summarize, the aforementioned studies depict a scenario in which ketamine preferentially inhibit GABAergic interneurons in the PFC, by blocking NMDARs or inhibiting the NRG1/ErbB4 signaling pathway. This leads to a transient downregulation of the inhibitory GABA tone, a subsequent glutamate surge, and thus an increase in the overall network activity. More questions regarding the exact mechanisms of the disinhibition hypothesis remain to be answered. For instance, GABAergic interneurons have been known to compose a variety of cell types, each with its unique protein expression profile, morphology, connection, and electrophysiological characteristics. Do these different types of interneurons contribute equally to the etiology of depression? Are they inhibited or modulated by ketamine to the same extent? Besides the PFC, will interneurons in other brain regions be potential target for antidepressant compounds?

### 4.2 BDNF/TrkB Signaling Pathway

Brain-derived neurotrophic factor (BDNF) belongs to a small family of secreted proteins such as nerve growth factor, neurotrophin 3 and neurotrophin 4. BDNF-mediated signaling pathway is acknowledged as a key regulator of neural circuit development and activity-dependent processes, which include neuronal differentiation and growth, synapse formation and plasticity, and higher cognitive functions (Duman and Monteggia, 2006; Park and Poo, 2013; Castren and Antila, 2017).

Systemic or local administration of BDNF exerts antidepressant-like effects (Shirayama, et al., 2002; Hoshaw et al., 2005; Schmidt and Duman, 2010), and over-expression of BDNF in the hippocampus renders animals resilient to chronic stress (Taliaz et al., 2011). It is proposed that essentially all antidepressant drugs promote activation of the high-affinity BDNF receptor tropomyosin receptor kinase B (TrkB) signaling, and this signaling is required for their behavioral effects (Duman and Monteggia, 2006; Autry and Monteggia, 2012; Castren, 2013; Castren and Antila, 2017). Earlier work demonstrated that, in the hippocampus, acute administration of systemic ketamine is associated with increased BDNF protein levels (Garcia, Comim et al., 2008), and a rapidly elevation of phosphorylated TrkB, an indicator of activated BDNF/TrkB signaling pathway (Autry et al., 2011). On the other hand, ketamine failed to exert antidepressant-like effects in transgenic mice with *BDNF* gene specifically knockdown in the forebrain (Autry et al., 2011), and infusion of BDNF neutralizing antibody into the mPFC abolished ketamine’s antidepressant-like responses in forced swim test (Lepack et al., 2014). Mice carrying human *BDNF* ^Val66Met^ single nucleotide polymorphism (SNP), which compromises BDNF processing and induces deficits in activity-dependent secretion of BDNF, failed to response to ketamine’s antidepressant-like action (Liu et al., 2012).

Recently, it is reported that ketamine binds to BDNF receptor TrkB in a stereoselective manner (Casarotto et al., 2021). By directly binding to a site formed by a dimer of TrkB transmembrane domains with a therapeutically relevant affinity, ketamine facilitates cell surface expression of TrkB and thus promotes BDNF/TrkB signaling mediated plasticity (Casarotto et al., 2021). Ketamine also inhibits the endocytosis of TrkB by disrupting the interaction of TrkB with the cargo-docking µ subunit of the AP-2 complex (Fred et al., 2019).

The effect of augmentation of BDNF/TrkB signaling by ketamine may not be limited in neuron cells. For instance, in C6 glioma cells, ketamine translocates Gα_s_ from lipid rafts to non-raft domains at clinically relevant concentrations (Wray et al., 2019). Ketamine-induced Gα_s_ redistribution leads to increased functional coupling of Gα_s_ and adenylyl cyclase (AC), and subsequent elevated intracellular cyclic adenosine monophosphate (cAMP) level. Increased cAMP level promotes phosphorylation of cAMP response element-binding protein (CREB), and subsequently upregulates BDNF expression in glia.

### 4.3 Synaptic Plasticity Regulated by Spontaneous NMDAR-Mediated Transmission

It has been hypothesized that spontaneous and evoked glutamatergic transmission are functionally segregated (Atasoy et al., 2008; Kavalali et al., 2011). At resting status, tonic neurotransmission of glutamate by spontaneous presynaptic vesicle release may activate NMDARs, and regulate synaptic plasticity and behavior.

Eukaryotic elongation factor 2 kinase (eEF2K), also known as calmodulin-dependent protein kinase III, belongs to the atypical alpha-kinase family. The activity of eEF2K is regulated by intracellular concentration of calcium and calmodulin. The primary downstream substrate of eEF2K is eEF2, and eEF2 pathway plays an important role in regulation of protein synthesis, synaptic plasticity and memory consolidation (Taha et al., 2013). Resting NMDAR activity causes sustained eEF2K activation, which phosphorylates eEF2, effectively halting the translation of BDNF. Acute NMDAR blockade by antidepressant dose of ketamine attenuates eEF2 phosphorylation, thus lifting the inhibition on BDNF translation (Autry et al., 2011). Antidepressant-like response to ketamine is abolished in eEF2 kinase knockout mice, and acute low dose of ketamine fails to augment BDNF protein level in hippocampus in these transgenic mice (Nosyreva et al., 2013).

A recent study also indicated that maintenance of basal level NMDAR function may be a key permissive factor required for the antidepressant-like effect of ketamine. Activation of NMDAR at rest (due to spontaneous/asynchronized vesicle release) and by glutamate surge (due to evoked/synchronized vesicle release generated by presynaptic action potential) may lead to distinct responses in postsynaptic cells. Reelin is a glycoprotein of the extracellular matrix, and is involved with cell migration, synaptogenesis and neural network activity modulation (Faini et al., 2021). Transgenic mouse models with deletion of Reelin or apolipoprotein E receptor 2 (ApoER2) abolished ketamine’s antidepressant-like action and compromised synaptic plasticity in the hippocampal CA1 region. Pharmacological manipulation indicated that Reelin-ApoER2-Src family kinases (SFK) pathway components may partially underlie the antidepressant-like action of ketamine (Kim et al., 2021b). NMDAR differentiate between.

### 4.4 mTORC1 Signaling Pathway

With a ubiquitously expression pattern in eukaryotic cell types, mechanistic target of rapamycin (mTOR) is a large (259 kDa), highly conserved, serine/threonine kinase which belongs to the phosphoinositide 3-kinase-related kinase family (Sabatini et al., 1999). mTOR signaling is mediated through two large biochemical complexes defined by their protein composition, known as mTOR complex 1 (mTORC1) and mTOR complex 2 (mTORC2), respectively (Lipton and Sahin, 2014). Studies have demonstrated that mTOR signaling is broadly involved in important cellular processes in both physiological and pathological conditions, which include translation, transcription, protein degradation, autophagy, cytoskeletal assembly and synaptic development and plasticity (Hoeffer and Klann, 2010; Lipton and Sahin, 2014).

#### 4.4.1 mTORC1-4E-BP Pathway

It has been documented that the antidepressant-like action of ketamine require activation of the mTORC1 signaling pathway (Li et al., 2010; Li et al., 2011; Fukumoto et al., 2019; Abdallah et al., 2020). Ketamine activates mTORC1 protein synthesis in the PFC and hippocampus (Li et al., 2010; Paul et al., 2014; Adaikkan et al., 2018; Abdallah et al., 2020). Antidepressant dose of ketamine rapidly and transiently activates the mTOR signaling, as evaluated by the levels of phosphorylated (activated) eukaryotic initiation factor 4E binding protein 1 (4E-BP1) and p70 ribosomal S6 protein kinase (S6K), two best-characterized substrates of mTORC1. On the contrary, the antidepressant-like response to ketamine is abolished by infusion of rapamycin, an allosteric inhibitor of mTORC1, into the PFC of rodents, as measured by forced swim test and learned helplessness (Li et al., 2010; Li et al., 2011).

However, in a recent randomized, double-blind study, a single dose of rapamycin pretreatment failed to block the acute antidepressant action of ketamine in depressed patients (Abdallah et al., 2020). The discrepancy with preclinical observations may be explained by rapamycin’s anti-inflammatory actions *via* an mTOR-dependent mechanism (Thomson et al., 2009), or the dosage used with peripheral route of administration. In animal studies, the same administration route of rapamycin fails to induce detectable mTOR activation by phosphorylation (Autry et al., 2011).

mTORC1 participates in cap-dependent initiation of mRNA translation via its downstream target 4E-BPs (Sonenberg and Hinnebusch, 2009). Following phosphorylation by mTORC1, 4E-BPs dissociate from eIF4E, and promote mRNA translation initiation (Lipton and Sahin, 2014). It has been hypothesized that ketamine activates mTORC1-4E-BP signaling in pyramidal excitatory neurons of the cortex (Miller et al., 2016; Workman et al., 2018). Genetic deletion of 4E-BPs abolished the antidepressant-like effects of ketamine as measured by animal models of forced swim test and novelty-suppressed feeding task (Aguilar-Valles et al., 2021). On CA1 pyramidal neurons, ketamine-induced changes in synaptic transmission were abolished by genetic deletion of 4E-BP2 selectively in GAD2 (encoding glutamic acid decarboxylase 65 (GAD65)) positive GABAergic interneurons, suggesting that previous theory should be modified to include both excitatory and inhibitory neurons (Aguilar-Valles et al., 2021). Rheb (Ras homolog enriched in brain), a Ras family small GTPase and a downstream substrate of tuberous sclerosis complex 1/2 (TSC1/2), activates the mTOR signaling pathway by stimulating the phosphorylation of S6K and 4E-BP1. NMDA receptor activation stimulates S-nitrosylation of glyceraldehyde-3-phosphate dehydrogenase (GAPDH), which promotes degradation of Rheb by ubiquitin-E3-ligase seven *in absentia* homolog (Siah1). Blockade of NMDARs by ketamine may augment the mTOR signaling pathway by stabilizing Rheb and subsequently activating mTOR signaling pathway (Harraz et al., 2016).

#### 4.4.2 PI3K/Akt/GSK-3/mTORC1 Pathway

Accumulating evidences have demonstrated an interaction between BDNF/TrkB and mTOR pathways in regulating synaptic plasticity. Activation of phosphatidylinositol 3 kinase (PI3K) by BDNF/TrkB induces translocation of Akt (protein kinase B) to plasma membrane (Reichardt, 2006). Alternatively, BDNF/TrkB stimulates the MEK-mitogen-activated protein kinase (MAPK)/extracelluar signal regulated kinase (ERK) signaling pathway. These two pathways converge on mTORC1 in the regulation of protein translation (Yoshii and Constantine-Paton, 2010). In the PFC and hippocampus, a single antidepressant dose of ketamine rapidly increased phosphorylation of Akt and ERK, accompanied by a fast and transient phosphorylation of mTOR (Li et al., 2010; Yang et al., 2013; Zhou et al., 2014b; Miller et al., 2014; Zhang et al., 2017).

Glycogen synthase kinase-3 (GSK3) is a master switch serine-threonine kinase implicated in major depressive disorder (Li and Jope, 2010; Beurel et al., 2011; Beurel et al., 2015). GSK3 is a downstream target of Akt, and can phosphorylate several components of the PI3K/AKT/mTOR signaling network, including Akt. Thus, GSK3 is functionally integrated with the PI3K-AKT-mTOR signaling pathway (Sengupta et al., 2010; Hermida et al., 2017; Evangelisti et al., 2020).

Inhibition of GSK3 by subanesthetic dose of ketamine is necessary for the rapid antidepressant-like action of ketamine. Knock-in mice carrying a constitutively active GSK3 are completely resistant to ketamine’s antidepressant-like action as measured by animal model of learned helplessness. Furthermore, acute administration of a high dose of lithium, a GSK3 inhibitor, produces antidepressant-like effect comparable to that of ketamine (Beurel et al., 2011). The inhibition of GSK3 might be mediated by activation of Akt, which deactivates GSK3 by phosphorylation. This hypothesis is supported by the finding that PI3K/Akt antagonists prevented ketamine-induced phosphorylation of GSK3, and abolished ketamine’s antidepressant-like actions (Zhou et al., 2014a).

### 4.5 Synaptogenesis and Structural Plasticity

In depressed patients, decreased volume of hippocampus and the prefrontal cortex (PFC) (subgenual and anterior cingulate cortex) are documented. Atrophy of principle neurons in these regions is reversible with successful treatment or discontinuation of stress exposure (MacQueen et al., 2008; MacQueen and Frodl, 2011; McEwen et al., 2015; McEwen et al., 2016). This phenomenon leads to the hypothesis that ketamine may alleviate depression by promoting synaptogenesis, thus antagonizing synaptic degeneration and atrophy of neurons induced by depression.

Ketamine administration increased the levels of the postsynaptic proteins PSD95 and GluR1, as well as the presynaptic protein synapsin I in the PFC of rats (Li et al., 2010). These effects lagged behind ketamine-induced activation of mTOR signaling and lasted for up to 72 h, suggesting that synaptogenesis and structural changes may mediate long-term antidepressant-like effect of ketamine. Blockade of mTOR signaling completely abolished ketamine-induced synaptogenesis and behavioral responses in animal models of depression, indicating that mTOR signaling may function as a mediator that transfers transient intracellular changes into sustained synapse strengthening by regulating local protein translation (Moda-Sava et al., 2019).

Recently, studies have provided some clues regarding the time course of neural structural remodeling triggered by ketamine. Within 2 h of administration, ketamine rapidly enhances the potential of activity-dependent synaptogenesis in the mPFC, and this effect precedes changes in spine density (Wu et al., 2021a). Within 12 h, ketamine selectively reverses stress-induced dendritic spine elimination in the mPFC, and the rescue of spinogenesis is necessary for the sustained antidepressant-like behavioral effects of ketamine (Moda-Sava et al., 2019). In another longitudinal study, two-photon microscopy imaging demonstrated that a single dose of ketamine increases dendritic spine density in the mPFC within 24 h after administration, and this effect lasts for up to 2 weeks (Phoumthipphavong et al., 2016). This is due to an elevated spine formation rate, whereas spine elimination rate remains unchanged.

Collectively, these evidences raise a possible scenario in which, initially after administration, ketamine enhances glutamate-evoked synaptic plasticity, which may lead to a transient and labile spinogenesis process. Subsequently, ketamine increases spine density by stabilizing newly generated spinogenesis and preventing stress-induced spine elimination simultaneously, thus exerting prolonged antidepressant-like effects.

### 4.6 AMPAR-Mediated Synaptic Transmission

α-amino-3-hydroxy-5-methyl-4-isoxazole-propionic acid receptors (AMPARs) are one type of the principle ionotropic transmembrane receptors responsible for fast synaptic neurotransmission at glutamatergic excitatory terminals (Derkach et al., 2007). Studies revealed that pretreatment with AMPAR antagonist NBQX abolished the antidepressant-like action of ketamine in animal models of forced swim test, tail suspension test, learned helplessness, novelty-suppressed feeding test, and stress-induced sucrose preference (Koike et al., 2011; Walker et al., 2013; Zhou et al., 2014b; Fukumoto et al., 2014). On the other hand, AMPAR agonist Cx546 enhanced the antidepressant-like effects of ketamine in forced swim test (Koike and Chaki, 2014; Zanos and Gould, 2018). These studies collectively highlighted the involvement of AMPAR activation in ketamine’s antidepressant-like action.

Ketamine increases AMPARs containing GluA1 and GluA2 subunits on the membrane of the hippocampus within 3 h after administration (Nosyreva et al., 2013), increases the levels of the postsynaptic proteins PSD95 and GluA1 in the PFC of rats (Li et al., 2010), and increases AMPARs containing GluA1 and/or GluA2 subunits at synaptosome fractions in the mPFC and the hippocampus within 24 h post-injection (Li et al., 2010; Zanos et al., 2016). These newly recruited AMPARs in postsynaptic sites may be utilized for subsequent synaptic strengthening. Electrophysiological recordings demonstrated that low doses of ketamine induce an enhancement of AMPAR-mediated synaptic transmission in the mPFC and hippocampus (Bjorkholm et al., 2015; El Iskandrani et al., 2015). On the contrary, ketamine fails to generate antidepressant-like response in mice lacking the *GluA2* gene, and ketamine-induced AMPAR-dependent synaptic potentiation of Schaffer collateral-CA1 synapses in hippocampal slices is abolished in *GluA2* knockout animals (Nosyreva et al., 2013). Activation of mTOR signaling by ketamine is completely blocked by AMPAR antagonist NBQX.

#### 4.6.1 (2*R*,6*R*)-HNK Enhances Presynaptic Release by Inhibition of mGluR2

Following systemic ketamine administration, (2*S*,6*S*;2*R*,6*R*)-HNK, are the major HNK metabolites found in human plasma and in rodent plasma and brain (Zarate et al., 2012). Earlier studies in rodents found that ketamine and norketamine exert anesthetic effects, but (2*S*,6*S*;2*R*,6*R*)-HNK do not (Leung and Baillie, 1986). However, the (*2S*,*6S*;*2R*,*6R*)-HNK metabolites, particularly the (2*R*,6*R*)-HNK stereoisomer, were found effective in inducing antidepressant-like behavioral and cellular responses in mice (Zanos et al., 2016). High dose of (2*R*,6*R*)-HNK only partially impaired NMDAR-miniature excitatory postsynaptic currents (mEPSCs) (Suzuki et al., 2017), and the efficacy of (2*R*,6*R*)-HNK to displace NMDAR antagonist MK-801 is low. Since ketamine is a well-documented NMDAR blocker, this phenomenon leads to the hypothesis that (2*R*,*6R*)-HNK may exert antidepressant-like effect via mechanisms independent of NMDAR inhibition (Lumsden et al., 2019).

At concentrations associated with antidepressant-like effect, (2*R*,6*R*)-HNK has been found to produce robust synaptic potentiation of excitatory synaptic transmission in hippocampal slices (Zanos et al., 2016). Using pharmacological and transgenic mouse lines, a recent study reported that, *via* inhibition of mGluR2 at presynaptic glutamate terminal, (*2R*,*6R*)-HNK selectively enhances excitatory synaptic transmission in the CA1 region of hippocampus through a concentration-dependent increase in glutamate release probability. (*2R*,*6R*)-HNK also increases synchronized high-frequency gamma oscillations, an *in vivo* marker of AMPAR-dependent neuronal excitation (Zanos et al., 2019). Importantly, the mGluR2-dependent antidepressant-like effects of (*2R*,*6R*)-HNK was not mediated by NMDAR inhibition (Zanos et al., 2019). This conclusion was further verified by another group (Riggs et al., 2020). Interestingly, this follow-up study reported that (*2R*,*6R*)-HNK induced presynaptic potentiation persists in the presence of tetrodotoxin (TTX) (Riggs et al., 2020), indicating that spontaneous, instead of evoked, vesicle release at presynaptic site may mediate the (*2R*,*6R*)-HNK induced presynaptic potentiation.

In rats, chronic stress-induced depression caused reduction in glutamate transmission in the ventrolateral periaqueductal gray (vlPAG). A single systemic injection of (2*R*,6*R*)-HNK reversed the diminished glutamate transmission and surface GluA1 expression (Adaikkan et al., 2018), and bath application of (2*R*,6*R*)-HNK increased the frequency and amplitude of mEPSCs in the vlPAG (Chou et al., 2018). A single injection of (2*R*,6*R*)-HNK impaired LTP and depressed AMPAR-mediated responses in the NAc, whereas NMDAR-mediated transmission in dopaminergic neurons in VTA remained unchanged (Yao et al., 2018). In hippocampus, bath application of (*2R*,*6R*)-HNK caused a rapid and persistent potentiation of AMPAR-mediated fEPSPs in Schaffer collateral terminals in CA1 of hippocampal slices in rats (Riggs et al., 2020). Another group reported that 1 week after administration, (2*R*,6*R*)-HNK attenuated AMPAR-mediated large-amplitude bursts in CA3 of hippocampal slices (Chen et al., 2020). The discrepancy may rise from differences in brain region investigated. More likely, transient presynaptic augmentation may lead to adaptive intracellular changes including metaplasticity with time elapsing.

#### 4.6.2 (2*R*,6*R*)-HNK Enhances Plasticity-Relevant Signaling Cascades

At postsynaptic neurons, (2*R*,6*R*)-HNK-induced AMPAR activation may lead to subsequent activation of plasticity- or synaptogenesis-relevant signaling cascades, such as BDNF/TrkB, eEF2, and mTORC1 pathways. These intracellular signaling pathways may also be recruited by ketamine, suggesting a partially overlapping mechanism of antidepressant-like action between ketamine and its metabolite (2*R*,6*R*)-HNK.

Infusion of (2*R*,6*R*)-HNK into the mPFC induces a rapid and long-lasting antidepressant-like effects in behavioral tests in mice. The antidepressant-like action of (2*R*,6*R*)-HNK is blocked in transgenic mice carrying knockin of the BDNF Val66Met allele which compromises the processing and activity-dependent release of mature BDNF. The antidepressant-like action of (2*R*,6*R*)-HNK is also abolished by anti-BDNF neutralizing antibody, or pharmacological inhibition of the components in the BDNF/TrkB signaling pathway such as L-type voltage-dependent Ca^2+^ channels (VDCCs), TrkB, and mTORC1 (Fukumoto et al., 2019). Recently, it is reported that (*2R,6R*)-HNK enhances BDNF/TrkB signaling by directly binding to TrkB (Casarotto et al., 2021), or by disrupting the interaction of TrkB with AP-2, thereby inhibiting TrkB endocytosis (Fred et al., 2019).

Collectively, these results indicate the modulation of AMPAR-mediated glutamate transmission as a possible mechanism mediating the antidepressant-like effects of (2*R*,6*R*)-HNK. In contrast to ketamine, the lack of robust NMDAR inhibition by (*2R*,*6R*)-HNK may underlie its lower adverse effect and abuse potential. This mechanism might be exploited for the development of novel antidepressants without side effects.

## Conclusion and Future Perspectives

Accumulating studies have yielded exciting insights and hypotheses regarding the underlying mechanism of the antidepressant-like effect of ketamine. In short, ketamine potentiates AMPAR-mediated transmission in pyramidal neurons and activates neuroplasticity- and synaptogenesis-relevant signaling pathways, which may converge on BDNF/TrkB and mTORC1. A net effect of these processes may be a transient potentiation of excitatory synaptic transmission, and in the long run, a dynamically sustained integrity of neural network structure and activity that might be compromised by depression (Moda-Sava et al., 2019, Wu et al., 2021a).

Changes in neural connectivity are becoming increasingly implicated in depression, so it is reasonable to consider that antidepressants are capable of producing structural changes over several weeks of continuous treatment. Besides protein translational modulation, transcriptional mechanism may be recruited as well. Following this thought, a recent paper gave us a first glimpse regarding how the antidepressant-like effect of ketamine is sustained beyond its pharmacokinetic time course. This study elegantly pinpointed methyl-CpG-binding protein 2 (MeCP2) as a key consolidating factor for the prolonged antidepressant-like effect of ketamine. Mice carrying a MeCP2 mutation abolished the persistant antidepressant-like action of ketamine, leaving its rapid antidepressant-like action intact (Kim et al., 2021a). MeCP2 is a key component of constitutive heterochromatin, and is crucial for chromosome maintenance and transcriptional silencing. Regarding how transient intracellular signaling is translated into sustained chromatin rearrangement and transcriptional modulation, the details remains elusive. Longitudinal comparison of transcriptional profiles after ketamine administration may shed new light on the mysterious link(s).

Ketamine and its metabolite (*2R*,*6R*)-HNK, when combined with other potential antidepressants, may exert synergistic effects by converging on certain pivotal intracellular processes. For instance, Glu2/3 receptor antagonists have been reported to increase glutamate release at excitatory terminals, mimicking the reported action of (*2R*,*6R*)-HNK (Zanos et al., 2019). AMPAR activation by presynaptic glutamate surge may trigger subsequent events, such as augmentation of BDNF/TrkB and mTORC1 signaling pathways, resulting in increased translation and transcription of proteins implicated in synaptic plasticity, and eventually leading to sustained structural alterations in neural circuit (Nishitani et al., 2014; Zanos et al., 2018b; Pham et al., 2018; Hashimoto, 2019; Riggs and Gould, 2021). These series of consequences may be shared by different antidepressants to some extent. For instance, GLYX-13 (also known as Rapastinel), a novel allosteric modulator of the NMDAR with glycine-like partial agonist properties, exerts rapid and sustained antidepressant-like actions *via* BDNF/TrkB signaling (Kato et al., 2018). In another case, selective serotonin 2C (5-HT2C) antagonists exert antidepressant-like actions, accompanied by induction of BDNF/TrkB, mTOR and eEF2 signaling in the mPFC (Opal et al., 2014). On the other hand, the similarity of antidepressant behavioral responses induced by different antidepressants might be exploited to help designing experiments in order to unravel the neurobiology of depression.

Structural analysis has negated a direct binding between ketamine and AMPARs at antidepressant relevant concentration (Zhang et al., 2021a). The exact mechanism via which ketamine enhances AMPAR-mediated transmission remains enigmatic. At postsynaptic site, as a well-documented NMDAR blocker, ketamine may diminish or even abolish NMDAR mediated-transmission, leading to synaptic depression; on the other hand, ketamine may augment AMPAR-mediated transmission, leading to synaptic potentiation. An interesting issue therefore rises regarding a possible crosstalk between these two major ionotropic glutamate receptors under the influence of ketamine. The net effect of ketamine on synaptic plasticity and homeostasis may differ, depending on factors such receptor subtype and location, relative abundance, and brain regions being investigated.

In this review, we mainly discussed investigations conducted in the PFC and hippocampus. However, other brain regions, such as VTA, NAc, PAG, thalamus, LHb, amygdala, have also been acknowledged to be implicated in depression. These regions have been reported to be reciprocally connected with the PFC. The heterogeneous connectivity within and among different nuclei may lead to their distinct responses to ketamine, thus adding another layer of complexity regarding the influence of ketamine. This possibility may be explored by combining pharmacological approach with optogenetic and chemogenetic tools at neural circuit level.

Here, we briefly mention several hypothesized mechanisms not covered in this review, these may function synergistically with the aforementioned mechanisms in mediating the antidepressant-like action of ketamine. Strict scrutinizes of these mechanisms may deepen our understanding about the etiology and pathophysiology of depression, and help translating mechanistic insights into clinical applications. Readers are encouraged to refer to some excellent reviews (Duman et al., 2016; Muir et al., 2019; Kavalali and Monteggia, 2020; Jelen et al., 2021; Jia et al., 2021; Riggs and Gould, 2021) for a more comprehensive coverage of these topics. Besides glutamatergic system, monoaminergic and opioid systems may also be remodeled by ketamine. Gut microbiota and immune system may also be regulated by ketamine due to its anti-inflammatory effects. Glia cell and extracellular matrix have been documented to be functionally integrated in synaptic plasticity, it is reasonable to speculate that ketamine may also exert antidepressant-like effects through glia.

Besides depression, ketamine induced synaptic plasticity and structural remodeling might be potentially beneficial for the treatment of other psychiatric disorders or neurological diseases, featured by altered connectivity and network function in the central nervous system. These diseases may include schizophrenia, bipolar disorder, anxiety, post-traumatic stress disorder (PTSD), neurodevelopmental disorder, cognitive decline and Alzheimer’s diseases.

The discovery of ketamine as a potential antidepressant with a rapid onset and sustained antidepressant-like actions revolutionized the concept about pharmacological interventions on depression. A full revealing of the underlying mechanisms of ketamine’s antidepressant-like actions precisely at molecular, cellular and neural circuit level, will not only deepen our understanding on the neurobiology of major depression, but may also expedite identification of potential therapeutic targets for the development of novel rapid-onset antidepressant drugs with favorable safety profile, sustained effects, and lacking undesired side effects.

## Abbreviations

AC, adenylyl cyclase; AMPAR, α-amino-3-hydroxy-5-methyl-4-isoxazolepropionic acid receptor; BDNF, brain-derived neurotropic factor; cAMP, cyclic adenosine monophosphate; EAAT2, excitatory amino acid transporter 2; 4E-BP1, eukaryotic initiation factor 4E binding protein 1; eEF2, eukaryotic elongation factor 2; eEF2K, eukaryotic elongation factor 2 kinase; ERK/MAPK, MEK-mitogen-activated protein kinase; GABAAR, gamma aminobutyric acid receptor, type A; GAPDH, glyceraldehydes-3-phosphate dehydrogenase; GSK, glycogen synthase kinase; mGluR2/3, metabotropic glutamate receptor 2/3; mTORC1, mechanistic target of rapamycin complex 1; NMDAR, N-methyl-D-aspartate receptor; NRG1, neuregulin; PI3K, phosphatidylinositol 3 kinase; Rheb, Ras homolog enriched in brain; S6K, p70 ribosomal S6 protein kinase; Siah1, seven in absentia homolog; TrkB, tropomyosin receptor kinase B; TSC2, tuberous sclerosis complex 2.
